# Engaging male partners in women's microbicide use: evidence from clinical trials and implications for future research and microbicide introduction

**DOI:** 10.7448/IAS.17.3.19159

**Published:** 2014-09-08

**Authors:** Michele Lanham, Rose Wilcher, Elizabeth T Montgomery, Robert Pool, Sidney Schuler, Rachel Lenzi, Barbara Friedland

**Affiliations:** 1 Social and Behavioral Health Sciences, FHI 360 Durham, NC, USA; 2 Research Utilization, FHI 360 Durham, NC, USA; 3 Women's Global Health Imperative, RTI International, San Francisco Project Office San Francisco, CA, USA; 4 Centre for Social Science and Global Health, University of Amsterdam, Amsterdam, The Netherlands; 5 HIV and AIDS Program, Population Council New York, NY, USA

**Keywords:** Microbicides, HIV, qualitative research, partner communication, gender relations

## Abstract

**Introduction:**

Constructively engaging male partners in women-centred health programs such as family planning and prevention of mother-to-child HIV transmission has resulted in both improved health outcomes and stronger relationships. Concerted efforts to engage men in microbicide use could make it easier for women to access and use microbicides in the future. This paper synthesizes findings from studies that investigated men's role in their partners’ microbicide use during clinical trials to inform recommendations for male engagement in women's microbicide use.

**Methods:**

We conducted primary and secondary analyses of data from six qualitative studies implemented in conjunction with microbicide clinical trials in South Africa, Kenya, and Tanzania. The analyses included data from 535 interviews and 107 focus groups with trial participants, male partners, and community members to answer research questions on partner communication about microbicides, men's role in women's microbicide use, and potential strategies for engaging men in future microbicide introduction. We synthesized the findings across the studies and developed recommendations.

**Results:**

The majority of women in steady partnerships wanted agreement from their partners to use microbicides. Women used various strategies to obtain their agreement, including using the product for a while before telling their partners, giving men information gradually, and continuing to bring up microbicides until resistant partners acquiesced. Among men who were aware their partners were participating in a trial and using microbicides, involvement ranged from opposition to agreement/non-interference to active support. Both men and women expressed a desire for men to have access to information about microbicides and to be able to talk with a healthcare provider about microbicides.

**Conclusions:**

We recommend counselling women on whether and how to involve their partners including strategies for gaining partner approval; providing couples’ counselling on microbicides so men have the opportunity to talk with providers; and targeting men with community education and mass media to increase their awareness and acceptance of microbicides. These strategies should be tested in microbicide trials, open-label studies, and demonstration projects to identify effective male engagement approaches to include in eventual microbicide introduction. Efforts to engage men must take care not to diminish women's agency to decide whether to use the product and inform their partners.

## Introduction

Microbicides were originally conceived of as a female-controlled HIV prevention method [1]. Subsequent research has found that although many women appreciate a product they can use without a partner's knowledge [2–7], microbicide trial participants typically talked with their steady partners about the product [3, 6, 8–12]. Moreover, male partners’ awareness and acceptance of product use influenced trial participants’ product acceptance and self-reported adherence [7, 13–16]. Male partner influence seems to differ by partner type, however. In several trials, women reported better adherence and greater ease using products discretely with casual, transactional, and non-cohabitating partners compared to steady partners [11, 16–18]. Involving male partners in microbicide use can also affect relationship quality; some trial participants reported that involving their partners improved partner communication and increased shared responsibility for HIV protection [13, 16, 19–21].

After more than two decades of testing microbicides in clinical trials, the CAPRISA 004 trial of 1% tenofovir gel provided proof of concept that microbicides can reduce women's HIV risk [22]. Ongoing clinical trials, including the FACTS 001 trial of 1% tenofovir gel and the MTN-020 and IPM 027 trials of the dapivirine ring, could provide sufficient evidence to support product licensure and introduction. Male engagement in family planning and prevention of mother-to-child HIV transmission (PMTCT) has been shown to improve acceptability, uptake, adherence, and health outcomes [23–27]. Considering whether and how to engage male partners at the outset of microbicide introduction could help maximize the potential for HIV prevention.

This paper synthesizes findings from microbicide studies that investigated men's role in their partners’ microbicide use during clinical trials in sub-Saharan Africa. Clinical trials are a unique environment, so men's roles may differ in the context of “real world” use. Nevertheless, the data provide a starting point for identifying effective ways to engage male partners in future research and eventual microbicide introduction.

## Methods

In January 2013, FHI 360 convened a meeting of microbicides experts and male engagement experts to develop the following research questions to inform recommendations for engaging male partners in microbicide introduction:

1. What existing gender norms could facilitate or hinder constructive male engagement in microbicide use?
2. What did women in the trials tell their male partners about the trial and/or the product, when did they tell them, and why?
3. How have men been involved in women's use of microbicides?
4. What information do men want about microbicides?
5. What are the best ways to engage men in women's microbicide use?
6. What are men's barriers to accompanying their partners to the clinic for microbicide services?

To answer the research questions, FHI 360 and the Kenya Medical Research Institute collected qualitative data in Kisumu, Kenya, where three microbicide trials had been conducted. FHI 360 also partnered with social scientists who had previously collected qualitative data about male engagement during microbicide trials.

This paper includes data from six qualitative studies (described in detail in Table 1). The trials all tested microbicide gel formulations, except IPM 015, which tested a microbicide ring. The VOICE trial tested oral pre-exposure prophylaxis as well as daily 1% tenofovir gel, and the mock clinical trial tested a proxy gel and proxy oral pill. All studies received approval from local ethics committees, and informed consent was obtained from each study participant.

**Table 1:** Qualitative data included in the analysis

Clinical trial	Qualitative data included in analysis
Trial: MDP301 [28] Phase: III Product: PRO2000 vaginal gel Length of product use: 12 months (24 in Uganda) Years: 2005–2009 Sites: 13 sites in South Africa, Tanzania, Uganda, and Zambia Participants: 9,385 sexually active women, aged 18 years or older (≥16 years in Tanzania and Uganda)	Study name: MDP301 [10, 29–31] Main focus: Social science sub-study to assess the accuracy of behavioural and adherence data, acceptability of the gel and the trial procedures, and understanding of the trial and the consent procedure Years: 2005–2009 Sites: 3 sites in Johannesburg, Durban, rural KwaZulu-Natal, South Africa Ethics approvals: Witwatersrand University Human Research Ethics Committee; University of KwaZulu-Natal Biomedical Research Ethics Committee; The Medicines Control Council of South Africa Participants: – 154 individual in-depth interviews (IDIs) with 90 men and women (45 couples). These women shared information about the trial and involved their partners from early on – 60 IDIs with 30 women who did not immediately inform their partners – 31 focus group discussions (FGDs) with trial participants, 18 FGDs with women in the community and 18 FGDs with men in the community Methods for secondary analysis: NVivo software was used to re-analyse IDIs and FGDs, through additional coding and extraction of themes relevant to male engagement

Trial: Carraguard Phase 3 Trial [32] Phase: III Product: Carraguard vaginal gel Length of product use: 9–24 months Years: 2004–2007 Sites: 3 sites in South Africa (Gugulethu, Soshanguve and Isipingo) Participants: 6,202 women, aged 16 and older	Study name: Evaluation of the Informed Consent Process in the Phase 3 Study of the Efficacy and Safety of the Microbicide Carraguard^®^ in Preventing HIV Seroconversion in Women [33–37] Main focus: Informed consent Years: 2006–2007 Sites: Gugulethu and Soshanguve, South Africa Ethics approvals: Population Council Institutional Review Board; Ethics Committee of the University of Cape Town; the Research Ethics and Publication Committee of the University of Limpopo, Medunsa Campus Participants: – 103 IDIs with trial participants – 5 FGDs with trial participants (*n*=29) – 1 mixed gender FGD (*n*=3 trial participants, 2 male partners) – 2 FGDs with male partners (*n*=8)
	Study name: Microbicides Acceptability: A Qualitative Study to Explore Social and Cultural Norms, Interpersonal Relations and Product Attributes [38, 39] Main focus: Sexual norms and gender roles affecting microbicide acceptability Years: 2006–2007 Sites: all three sites Ethics approvals: Population Council Institutional Review Board; Ethics Committee of the University of Cape Town; the Research Ethics and Publication Committee of the University of Limpopo, Medunsa Campus; the Biomedical Research Ethics Committee, University of Kwa-Zulu Natal Participants: – 62 IDIs with trial participants – 14 FGDs with trial participants (*n*=97) – 2 FGDs with male partners (*n*=13) – 3 IDIs with male partners Methods for secondary analysis: Thematic analysis of the previously coded data was conducted for codes most pertinent to the research questions

Trial: MTN-003 “VOICE” [40] Phase: IIb Product: Vaginal 1% tenofovir gel, oral tenofovir, oral tenofovir/emtricitabine	Study name: MTN-003C “VOICE-C” [41] Main focus: Household and community-level factors associated with study product adherence in VOICE Years: 2010–2012
Length of product use: Up to 36 months Years: 2008–2012 Sites: South Africa (3 sites), Zimbabwe, and Uganda Participants: 5029 women, half were aged 18–24	Site: Johannesburg, South Africa Ethics approvals: Office of Research Protection Institutional Review Board, RTI International; Human Research Ethics Committee of the University of the Witwatersrand Participants: – 22 male partners in 14 IDIs and 2 FGDs (*n*=8) – 102 randomly-selected female VOICE participants in IDIs (*n*=41), ethnographic interviews (*n*=21) and 7 FGDs (*n*=40) Methods for secondary analysis: Thematic analysis of coded transcripts from all IDI, EI and FGD data from male partners and female study participants described above

Trial: IPM 014A Phase: I/II Product: Dapivirine vaginal gel 4759, 0.05% 2.5G Length of product use: 6 weeks Years: 2009 Sites: Kenya, Malawi, Rwanda and South Africa Participants: Approximately 320 women, aged 18–40 Trial: IPM 015 Phase: I/II Product: Dapivirine vaginal ring Length of product use: 12 weeks Years: 2010 Sites: Kenya, Malawi, Rwanda, South Africa and Tanzania Participants: Approximately 280 women, aged 18–40	Study name: Male Engagement in Microbicides Main focus: Identifying strategies for engaging men in future trials, open-label studies, demonstration projects, and microbicide introduction so they will support their female partners in using microbicide products for HIV prevention or, at least, to minimize men's interference in women's microbicide use Years: 2013 Site: Kisumu, Kenya Ethics approvals: Kenya Medical Research Institute Ethical Review Committee; FHI 360's Protection of Human Subjects Committee Participants: – 30 IDIs with former female trial participants – 25 IDIs with women who were not trial participants – 14 IDIs with men who were partners of trial participants at the time of the trial – 29 IDIs with men who were not partners of trial participants Methods for analysis: Thematic analysis of IDIs using NVivo 9 software. The saliency of themes was assessed via frequencies, degrees of emphasis and elaboration, co-occurrences, and contrasts of themes across interviews, and compared between participant groups
Trial: MTN-004/ VivaGel [42] Phase: I Products: VivaGel^®^ (SPL7013 gel) vaginal gel Length of product use: 14 days Years: 2006–2007 Sites: United States, Kenya Participants: 54 sexually active women, aged 18 to 24	

Trial: Adolescents and Microbicide Clinical Trials: Assessing the Opportunities and Challenges of Participation [43–46] Phase: Mock clinical trial Product: Proxy vaginal gel (Pre-Seed lubricant) and proxy oral pill (Vitacap multivitamin) Length of product use: optional 0–2 months Years: 2011–2013 Sites: Tanzania Participants: 135 sexually active adolescents and young women, aged 15–21	Study name: Adolescents and Microbicide Clinical Trials: Assessing the Opportunities and Challenges of Participation [45, 47–49] Main focus: Socio-cultural factors that hinder young women's participation in topical or oral microbicide trials Years: 2010–2011 Site: Dar es Salaam, Tanzania Ethics approvals: Muhimbili University of Health and Allied Sciences; National Institute for Medical Research (Tanzania); FHI 360's Protection of Human Subjects Committee Participants: – 3 FGDs with mothers of adolescents in the community (*n*=25) – 2 FGDs with fathers of adolescents in the community (*n*=18) – 2 FGDs with unmarried male partners of adolescents in the community (*n*=14) Methods for secondary analysis: Thematic analysis of coded excerpts from all FGD data from male partners, fathers, and mothers of adolescent girls

Social scientists from each study conducted primary or secondary analyses of their data and summarized the findings to answer the research questions, and FHI 360 staff synthesized the findings across the six studies. In November 2013, the social scientists and additional experts met to discuss each study's results and identify central themes and programmatic implications of the data.

## Results

### Gender norms

Prevailing gender norms about sexuality and complex relationship dynamics affected how women addressed their trial participation and microbicide use with male partners. Couples were more likely to discuss HIV risk, get tested, and use condoms at the beginning of a relationship. Such discussions were considered less acceptable later in a relationship. For some men, a partner's use of microbicides was a sign that she suspected he was unfaithful or that she had outside partners. Women found ways to work within the existing patriarchal gender relations, such as negotiating microbicide use without openly challenging male authority or voicing suspicions of infidelity. In some cases men and women implied that men needed a public appearance of authority, but were more willing to be flexible within the privacy of their relationships.

### Partner communication

Women and men agreed that, ideally, women would discuss microbicide use with their steady partners. The majority of women wanted agreement from their steady partners to use microbicides, though many women and some men thought it was ultimately the woman's decision. Trial participants typically decided whether to discuss microbicide use with their partners based on: 1) the nature of their relationship, 2) the partner's temperament, and 3) evaluation of how the partner might react if told in advance versus finding out the product had been used without his knowledge. Though microbicide use and trial participation are distinct topics, women usually did not distinguish between the two when communicating with partners.

Women gave two main reasons for not discussing microbicide use with partners. They either feared a negative reaction, ranging from mere objection to violence, or thought their partners did not need to know, particularly in casual relationships. Women who feared a negative reaction often thought the benefit of using microbicides was worth the risk of their partners later discovering their covert use. Contrary to the widely reported belief that discreet use would cause problems, some men who were unaware of their partner's microbicide use were not upset when they found out about it.

Trial participants who decided to discuss microbicides with their partners did so for emotional, logistical, and strategic reasons. Many women said they wanted to promote an open, trusting relationship or prevent a disagreement or breakup. Others wanted to gain the partner's support in case they later experienced side effects or other problems. Some felt it was “the right thing to do,” because their partners would also be exposed to an experimental product, and others thought it would be difficult to explain the sudden need to use condoms, frequent visits to the clinic, and (for gel users) change in lubrication during sex. They also said it would be challenging to hide the applicators and insert the gel. Women reported a stronger sense of desire or obligation to discuss microbicide use in steady relationships than casual relationships. They also said that the logistics of trial participation and product use were more difficult to keep secret from a steady partner.

The vast majority of women in MDP301 and many women in the other studies told their partners about their microbicide use early during trial participation. The follow-up study in Kenya was an exception; one-third of the women interviewed said they had not told their partners. This may have been because they used the product for a shorter period of time compared to women in the other studies, making it less necessary to inform their partners.

When male partners were initially resistant to using microbicides, women used a number of strategies to obtain their agreement. Some women used the product for a while before telling their partners about it. If a partner objected, the woman reminded him that he had not noticed a difference, or in some cases that he had noticed but was enjoying their sexual experience. This was particularly the case with microbicide gels; many couples found the lubrication from the gel increased pleasure or at least decreased pain. Other women gave their partners incomplete information (e.g. not mentioning that the microbicide contained an antiretroviral drug) or misinformation (e.g. saying it was a family planning method). Other strategies included giving men information gradually or continuing to bring up microbicides until resistant partners acquiesced. Some women made their partners feel that they were making the decision, playing into the gender norm that men dominate decision-making. When partners did not agree on microbicide use, some women succeeded in using without their knowledge. See Table 2 for illustrative quotes about partner communication.

**Table 2:** Select themes and illustrative quotes

Theme	Illustrative quotes
**Reasons women discussed microbicides with their partners**	
To promote an open, trusting relationship or to prevent a disagreement or breakup	*If we did not have an agreement before I started participating in the study, […] maybe we would have fought and I would have decided to quit the study. So I tell him about a lot of things before they happen because I don't like keeping secrets*. (VOICE participant, Johannesburg, South Africa)
To gain their partner's support in case they later experienced problems	*I thought I should tell them [partner and a friend] because maybe if I tell them […] and there are problems while I am participating at least they will know that I am in the trial. […] They can give me support*. (Carraguard participant, Soshanguve, South Africa)
Would be difficult to hide trial participation and microbicide use	*I had to because it was going to be difficult to do anything without telling him. He was going to be surprised if I start applying the gel and he does not know about it, it was not going to be possible*. (Carraguard participant, Soshanguve, South Africa)

**Reasons women did not discuss microbicides with their partners**	
Feared a negative reaction	*My partner is a difficult guy to deal with …. He's noisy, violent, and anything you tell him he disapproves; even if I left the house for a few minutes he would think that I've gone to see other men …. He would have beaten me up or chased me away …. I think it should be a secret*. (IPM 014A participant, Kisumu, Kenya)
Thought their partners did not need to know	*It just did not come to my mind to tell him because I think it is too early in our relationship. I will tell him later*. (VOICE participant, Johannesburg, South Africa)

**Strategies used with reluctant partners**	
Used the product for a while and then told partner about it	*I told him about it and he refused to allow me to use the gel. […] I said to him, ‘it's fine’ … [but] I used the gel without telling him and we had sex all the time. […] He did not feel it. And after two months using it in secret I talked to him again about it. But I did not tell him I was using it in secret. Then he said that if I used the gel he would not have sex with me. I asked him why. He said that he did not know if sex was going to be the same or not. Then I asked him how he felt about our sex and he said that it was okay. I then told him I had been using the gel, and he said that I could continue to use it*. (MDP301 participant, South Africa)
Gave partners incomplete information or misinformation	*I just told him that I was participating in a study*. (IPM 015 participant, Kisumu, Kenya) *I just told him we were studying something on family planning*. (IPM 015 participant, Kisumu, Kenya)
Gave partner information gradually or continuing to bring up microbicides with their resistant partner until they acquiesced	*I told my husband that at Empilisweni [study clinic] it is said there is a gel that is being used that helps prevent HIV; my husband refused that I come here …. When I came home I told him that I had been here and I watched a video and I'm going to come again, and he wasn't agreeable, resisting, and I came again …. and then on another day he gave me permission*. (Carraguard participant, Gugulethu, South Africa)

**Continuum of male engagement in women's microbicide use**	
Opposition	*Personally if you ask me I will not allow her because of one reason, I think she can be like a slave; every end of month she will be attending clinic. Also, she will not be allowed to carry pregnancy; it means we will postpone getting children because of that study*. (Male partner of adolescent woman, Dar es Salaam, Tanzania)
Agreement/non-interference	*… he had the option of stopping me from using the product. He will tell me not to participate in anything that he doesn't like. I went to the clinic and he knew about it. He never stopped me. That means he supported my decision*. (IPM 015 participant, Kisumu, Kenya) *When she comes here she tells me that she is coming here [study clinic], I do not have a problem and I allow her to come here*. (Male partner of VOICE participant, Johannesburg, South Africa) *I have told her one thing ‘if you are happy and it is treating you well [not reacting negatively to you], I do not have a problem, and I will not follow you or accompany you when you go to the study because to a certain extent [if I do that] you may think that I do not trust you.’* (Male partner of VOICE participant, Johannesburg, South Africa)
Active support	*[My partner] was very receptive. He even accompanied me to the clinic when he was off duty. Sometimes he even ask me before we have sex that ‘are you not applying the gel, please don't forget it.’* (Carraguard participant, Soshanguve, South Africa) *I used to give her money for clinic visits and took care of our children when she went to the clinic*. (IPM 014A participant, Kisumu, Kenya)

### Men's roles

**Figure 1:**
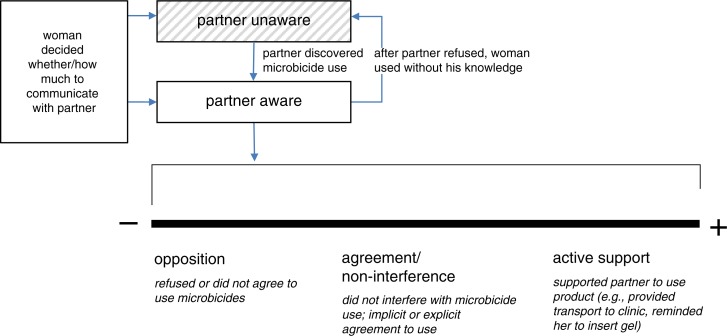
Continuum of male partner involvement in microbicide use.

Among men who knew about their partner's participation in a trial and use of microbicides, involvement ranged from opposition to agreement/non-interference to active support (See Figure 1, Table 2). Most men fell in the “agreement/non-interference” part of the continuum. This may not be representative of all trial participants’ experiences, because women who encountered opposition from their partners may have discontinued trial participation or elected not to participate in interviews.

#### Opposition

Some men were opposed to their partners participating in the trial and/or using microbicides because they had concerns about product safety, potential partner infidelity, and the researchers’ intent. Some men were resistant because they lacked knowledge about the product or trial, and some did not want their partners participating in something beyond their control or understanding. Their opposition ranged from voicing unease or uncertainty to outright refusal.

#### Agreement/non-interference

Some men gave their partners permission to use microbicides. In other cases, male partners’ “agreement” was tacit; they did not give express permission, but knew their partners were using microbicides and did not interfere.

#### Active support

Some men provided their partners with instrumental or emotional support to participate in a trial and use microbicides. They reminded their partners to use the gel or in some cases inserted it for them as part of foreplay. They helped their partners attend clinic visits by reminding them of their appointments, occasionally accompanying them, or giving them transport money. The studies did not quantify the proportion of men who provided these more active forms of support, though it seems they were in the minority. A larger number of men were reported to have adapted their sexual practices to meet the trial requirements of regular condom and microbicide use.

Men's involvement often varied over time. For example, some men were opposed to microbicide use at first, but after their partners convinced them to try it, they liked it and agreed to the product's use. Other men were generally supportive of microbicide use but occasionally suggested sex without the gel.

### Other considerations for engaging male partners

Periodic HIV counselling and testing (HCT) is required during trial participation and will be part of the service delivery package for any antiretroviral-based microbicide introduced in the public sector. Trial participation gave women a reason to discuss HIV testing and results with their partners. Indeed, men equated clinical trials with access to HIV testing and results, which elicited two common responses. A number of men were pleased their partners were getting tested regularly and being found to be HIV negative. Particularly in the studies in South Africa, men were generally unwilling to get tested themselves and used their partner's HIV-negative status as a proxy for their own status. Men in the study in Kenya were generally more open to HIV testing. The second common reaction was fear. Some men did not want to go to the trial clinic with their partners because they feared being forced to get tested.

Men and women expressed a desire for men to have access to information about microbicides. Men wanted to know about the safety and side effects of the product, whether microbicides prevent pregnancy, whether microbicides protect men from HIV, and whether they affect fertility or the sexual experience. Some also had questions (and suspicions) about the clinical trial process, including why it was being tested by “whites” or “foreigners” on African women.

Some women and men said it would be helpful if male partners could talk directly with a healthcare provider about microbicides, because providers carry authority and could legitimize the product and the research. Some felt their own ability to convey information in the most accurate and persuasive way was limited and wanted assistance from providers.

Several of the studies experienced challenges recruiting men for interviews and focus groups. Men were often unavailable because they were working. Others were hesitant to go to the study clinic because they were afraid it would involve HIV testing or thought “the clinic was meant for women only.” Some studies had more success recruiting men if they conducted interviews and focus groups outside of the clinic.

## Discussion

Findings from these six qualitative studies confirm that male partners play an important role in women's microbicide use and provide insights for engaging men in future microbicide studies and product introduction. Promoting women's agency to decide whether to use microbicides and inform their partners is of paramount importance [50]. Not only is each woman best equipped to understand whether it is safe and/or will be productive to discuss microbicide use with her partner, she is also likely to be most skilled in introducing the subject and ultimately gaining his support. Enabling women to make decisions about microbicide use and providing the skills and enabling environment to act on them will help ensure that microbicides, if effective, are the game-changer for women's HIV prevention they were intended to be.

We recommend the primary goal of male engagement in women's microbicide use be to promote women's ability to access and use the product. To accomplish the primary goal, we recommend 1) providing support to women through counselling to decide whether and how to involve their partners and 2) sharing basic information about microbicides with men through couples’ counselling, community education, and mass media to increase their awareness and acceptance of the product (see Table 3).

**Table 3:** Recommended strategies for engaging male partners

Goal	Objectives	Strategies	Rationale	Considerations
Increase women's ability to access and use microbicides	Support women to decide whether/how to talk with partner about microbicides	Counselling for women	Context of trial participation provided women supportive environment for communicating with partner; women will need support in real world introduction	Include: – Women's right to decide whether to talk with her partner – Strategies for communicating with partner to gain his approval – Strategies for using microbicides without partner's knowledge – Written materials women can share with partner
	Increase men's awareness and acceptance of microbicides to create an enabling environment for women to discuss and negotiate microbicide use	Couples counselling on microbicides	Women and men expressed a desire for men to be able to talk with health providers	Men hesitant to attend clinics because: – Feared HIV testing – Some clinics viewed as women's spaces – Scheduling conflicts To address these barriers: – Consider offering microbicides at clinics with services for men and women – Providers offer to call partners or visit homes
		Community education	May be more effective than reaching men through clinics	– Provide general information on benefits, safety – Target spaces where men congregate
		Mass media	May be more effective than reaching men through clinics	– Feature health providers, who are viewed as reliable sources of information – Promote benefits, safety – Show steady couples using microbicides

Some of these male engagement strategies – including counselling women, inviting male partners to the study clinic, offering couples counselling and informational materials, and doing community outreach to men – are currently being implemented at some microbicide trial sites to improve participant recruitment, retention, and adherence. However, the positive and negative effects of these approaches have not been rigorously evaluated. Consistent product use, which is crucial to effectiveness, has been lower than anticipated in trials [22, 32, 51], and may be improved through strategies to increase or improve positive male engagement. The male engagement strategies we recommend should be tested as part of a multi-pronged adherence support program. Their effect on partner communication, relationship quality and intimate partner violence should also be measured.

### Limitations

There are several limitations to our synthesis. Women participating in clinical trials may differ from women in the general population; they may be more empowered or more motivated to prevent HIV or may have partners who are more open to their participation, among other differences. Moreover, some of the qualitative studies experienced difficulty recruiting men; those who agreed were likely more supportive partners and therefore not representative of all male partners of trial participants. It is likely that some women did not participate in microbicide trials because of partner opposition, so such couples would not be represented in the studies. Clinical trials also provide a supportive context for women to discuss microbicides with their partners. Specifically, trial requirements give women leverage to negotiate condom and microbicide use with partners. In addition, women receive intensive counselling and support from trial staff. These conditions may be difficult to replicate in real world microbicide introduction, where providers have less time and fewer resources. Partner engagement may differ outside of clinical trials; women may be more or less likely to talk with their partners when they are using a product with known effectiveness and when condom use is recommended but not required.

Most of the studies focused on microbicide gels. Partner interaction may differ somewhat for other microbicide formulations, such as rings, films, suppositories, and injectables. For example, women using a gel may be more likely to tell their partners about their microbicide use compared to women using a ring, because they anticipate the increased lubrication caused by the gel will be noticed by partners or that it will be difficult to insert gel regularly without a partner's knowledge. Also, women may find it easier to talk with partners about use of a multipurpose microbicide product that protects against both HIV and pregnancy, because contraceptive use may be more acceptable than using HIV prevention methods in steady relationships.

Although our findings are not necessarily generalizable to microbicide use outside of the trial context or to different microbicide formulations, this is the first study to synthesize data on partner interactions in multiple clinical trials, and our recommendations provide a starting point for future male engagement efforts in microbicide research and programs.

## Conclusions

Microbicides by themselves will not alter the underlying gender norms that put women at risk of HIV. If proven effective, they may give women some level of increased control over HIV prevention, but many women – particularly those in steady relationships – may find it easier to access and adhere to a microbicide product if their male partners are supportive of its use. The strategies we recommend – based on data from multiple sites in three countries – could increase men's awareness and acceptance of microbicides, thereby potentially enhancing women's ability to access and use microbicides. While the strategies will need to be tailored to individual communities and specific microbicide formulations, they must consistently preserve women's agency to decide whether to use the product and inform their partners. They should be tested in microbicide trials, open-label studies, and demonstration projects to determine their impact on women's microbicide use and gender relations and identify effective male engagement strategies to include in eventual microbicide introduction.

## References

[1] Stein ZA (1990). HIV prevention: the need for methods women can use. Am J Public Health.

[2] Whitehead SJ, McLean C, Chaikummao S, Braunstein S, Utaivoravit W, van de Wijgert JH (2011). Acceptability of Carraguard vaginal microbicide gel among HIV-infected women in Chiang Rai, Thailand. PLoS One.

[3] Pool R, Whitworth JA, Green G, Mbonye AK, Harrison S, Wilkinson J (2000). An acceptability study of female-controlled methods of protection against HIV and STDs in south-western Uganda. Int J STD & AIDS.

[4] Hoffman S, Morrow KM, Mantell JE, Rosen RK, Carballo-Dieguez A, Gai F (2010). Covert use, vaginal lubrication, and sexual pleasure: a qualitative study of urban U.S. Women in a vaginal microbicide clinical trial. Arch Sex Behav.

[5] Rosen RK, Morrow KM, Carballo-Dieguez A, Mantell JE, Hoffman S, Gai F (2008). Acceptability of tenofovir gel as a vaginal microbicide among women in a phase I trial: a mixed-methods study. J Women's Health.

[6] van der Straten A, Montgomery ET, Cheng H, Wegner L, Masenga G, von Mollendorf C (2012). High acceptability of a vaginal ring intended as a microbicide delivery method for HIV prevention in African women. AIDS Behav.

[7] Montgomery ET, Cheng H, van der Straten A, Chidanyika AC, Lince N, Blanchard K (2010). Acceptability and use of the diaphragm and Replens lubricant gel for HIV prevention in Southern Africa. AIDS Behav.

[8] Gafos M, Mzimela M, Sukazi S, Pool R, Montgomery C, Elford J (2010). Intravaginal insertion in KwaZulu-Natal: sexual practices and preferences in the context of microbicide gel use. Cult Health Sex.

[9] Green G, Pool R, Harrison S, Hart GJ, Wilkinson J, Nyanzi S (2001). Female control of sexuality: illusion or reality? Use of vaginal products in south west Uganda. Soc Sci Med.

[10] Montgomery CM, Gafos M, Lees S, Morar NS, Mweemba O, Ssali A (2010). Re-framing microbicide acceptability: findings from the MDP301 trial. Cult Health Sex.

[11] Sahin-Hodoglugil NN, van der Straten A, Cheng H, Montgomery ET, Kacanek D, Mtetwa S (2009). Degrees of disclosure: a study of women's covert use of the diaphragm in an HIV prevention trial in sub-Saharan Africa. Soc Sci Med.

[12] Woodsong C (2004). Covert use of topical microbicides: implications for acceptability and use. Int Fam Plan Perspect.

[13] Montgomery ET, van der Straten A, Chidanyika A, Chipato T, Jaffar S, Padian N (2011). The importance of male partner involvement for women's acceptability and adherence to female-initiated HIV prevention methods in Zimbabwe. AIDS Behav.

[14] Salter ML, Go VF, Celentano DD, Diener-West M, Nkhoma CM, Kumwenda N (2008). The role of men in women's acceptance of an intravaginal gel in a randomized clinical trial in Blantyre, Malawi: a qualitative and quantitative analysis. AIDS Care.

[15] Mngadi KT, Maarschalk S, Grobler AC, Mansoor LE, Frohlich JA, Madlala B (2014). Disclosure of microbicide gel use to sexual partners: influence on adherence in the CAPRISA 004 trial. AIDS Behav.

[16] Greene E, Batona G, Hallad J, Johnson S, Neema S, Tolley EE (2010). Acceptability and adherence of a candidate microbicide gel among high-risk women in Africa and India. Cult Health Sex.

[17] Muchomba FM, Gearing RE, Simoni JM, El-Bassel N (2012). State of the science of adherence in pre-exposure prophylaxis and microbicide trials. J Acquir Immune Defic Syndr.

[18] Woodsong C, MacQueen K, Amico KR, Friedland B, Gafos M, Mansoor L (2013). Microbicide clinical trial adherence: insights for introduction. J Int AIDS Soc.

[19] Woodsong C, Macqueen K, Namey E, Sahay S, Morar N, Mlingo M (2006). Women's autonomy and informed consent in microbicides clinical trials. J Empir Res Hum Res Ethics.

[20] Pistorius AG, van de Wijgert JH, Sebola M, Friedland B, Nagel E, Bokaba C (2004). Microbicide trials for preventing HIV/AIDS in South Africa: phase II trial participants’ experiences and psychological needs. SAHARA J.

[21] Marlow HM, Tolley EE, Kohli R, Mehendale S (2010). Sexual communication among married couples in the context of a microbicide clinical trial and acceptability study in Pune, India. Cult Health Sex.

[22] Abdool Karim Q, Abdool Karim SS, Frohlich JA, Grobler AC, Baxter C, Mansoor LE (2010). Effectiveness and safety of tenofovir gel, an antiretroviral microbicide, for the prevention of HIV infection in women. Science.

[23] Barker G, Ricardo C, Nascimento M (2007). Engaging men and boys in changing gender-based inequity in health: evidence from programme interventions.

[24] Shattuck D, Kerner B, Gilles K, Hartmann M, Ng'ombe T, Guest G (2011). Encouraging contraceptive uptake by motivating men to communicate about family planning: the Malawi Male Motivator project. Am J Public Health.

[25] Becker S (1996). Couples and reproductive health: a review of couple studies. Stud Fam Plan.

[26] World Health Organization (2012). Male involvement in the prevention of mother-to-child transmission of HIV.

[27] Aluisio A, Richardson BA, Bosire R, John-Stewart G, Mbori-Ngacha D, Farquhar C (2011). Male antenatal attendance and HIV testing are associated with decreased infant HIV infection and increased HIV-free survival. J Acquir Immune Defic Syndr.

[28] McCormack S, Ramjee G, Kamali A, Rees H, Crook AM, Gafos M (2010). PRO2000 vaginal gel for prevention of HIV-1 infection (Microbicides Development Programme 301): a phase 3, randomised, double-blind, parallel-group trial. Lancet.

[29] Montgomery CM, Pool R (2011). Critically engaging: integrating the social and the biomedical in international microbicides research. J Int AIDS Soc.

[30] Montgomery CM, Watts C, Pool R (2012). HIV and dyadic intervention: an interdependence and communal coping analysis. PLoS One.

[31] Pool R, Montgomery CM, Morar NS, Mweemba O, Ssali A, Gafos M (2010). A mixed methods and triangulation model for increasing the accuracy of adherence and sexual behaviour data: the Microbicides Development Programme. PLoS One.

[32] Skoler-Karpoff S, Ramjee G, Ahmed K, Altini L, Plagianos MG, Friedland B (2008). Efficacy of Carraguard for prevention of HIV infection in women in South Africa: a randomised, double-blind, placebo-controlled trial. Lancet.

[33] Abbott S, Friedland B, Katzen L Health concerns and HIV risk perception as motives to join a clinical trial.

[34] Abbott S, Friedland B, Katzen L, de Kock A, Madiba S, Cishe S Decision making and the influences of others in the informed consent process: a qualitative investigation in the Phase 3 study of Carraguard^®^.

[35] Abbott S, Friedland B, Katzen L, Madiba S, de Kock A, Cishe S What motivates women to enroll in microbicide clinical trials: results from the Phase 3 Carraguard^®^ Trial.

[36] Katzen L, Friedland B, Abbott S, de Kock A, Madiba S, Cishe S Qualitative evaluation of the informed consent process in the Phase 3 Carraguard^®^ Efficacy Trial: study staff experiences and recommendations for future trials.

[37] Madiba S, Friedland B, Abbott S, Katzen L, de Kock A, Cishe S Male partner involvement in the Carraguard^®^ Phase 3 Trial: data from a qualitative study of the informed consent process.

[38] Abbott S, Morar N, Madiba S, Katzen L, Phillip J, Mokgatle-Nthabu M Microbicides acceptability: the influence of social and cultural norms, interpersonal relations and sexual socialization.

[39] de Kock A, Abbott S, Morar N, Friedland B, Mtimkulu V, Cishe S Exploring microbicide acceptability in terms of socio-cultural norms, relationships and product attributes in the Carraguard^®^ Phase 3 Trial in South Africa.

[40] Marrazzo J, Ramjee G, Nair G, Palanee T, Mkhize B, Nakabiito C Pre-exposure prophylaxis for HIV in women: daily oral tenofovir, oral tenofovir/emtricitabine or vaginal tenofovir gel in the VOICE study (MTN 003).

[41] van der Straten A, Stadler J, Montgomery E, Hartmann M, Magazi B, Mathebula F (2014). Women's experiences with oral and vaginal pre-exposure prophylaxis: the VOICE-C qualitative study in Johannesburg, South Africa. PLoS One.

[42] Cohen CR, Brown J, Moscicki AB, Bukusi EA, Paull JR, Price CF (2011). A phase I randomized placebo controlled trial of the safety of 3% SPL7013 Gel (VivaGel(R)) in healthy young women administered twice daily for 14 days. PLoS One.

[43] Headley J, Baumgartner JN, Kaaya S, Minja A, Kalungula H, Bangapi D (2014). Adolescent and young women's acceptability and use of a proxy microbicide gel or pill in a mock trial in Tanzania.

[44] Tolley E, Kaaya S, Kaale A, Kalungula H, Headley J, Baumgartner JN Do adolescents under 18 years old warrant inclusion in HIV prevention clinical research? Comparing sexual risk patterns of adolescent and young adult women in a mock trial in Tanzania.

[45] Baumgartner JN, Kaaya S, Kaale A, Headley J, Tolley E Domestic violence among adolescents and young women in HIV prevention research in Tanzania: participant experiences and measurement issues.

[46] Tolley E, Kaaya S, Bangapi D, Minja A, Headley J, Kaale A Feasibility of recruiting adolescent women into a mock HIV prevention trial.

[47] Tolley E, Baumgartner JN, Kaaya S, Sastry J, Headley J Adolescent sexual risk and perceived need for PrEP: paving the way for their future participation in HIV prevention trials.

[48] Baumgartner JN, Tolley E, Kaaya S, Sastry J, Headley J Adolescent and community perspectives on microbicide trial participation in Tanzania and India.

[49] Sastry J, Tolley E Research on adolescent sex: to do or not to do.

[50] Venables E, Stadler J (2012). ‘The study has taught me to be supportive of her’: empowering women and involving men in microbicide research. Cult Health Sex.

[51] Padian NS, van der Straten A, Ramjee G, Chipato T, de Bruyn G, Blanchard K (2007). Diaphragm and lubricant gel for prevention of HIV acquisition in southern African women: a randomised controlled trial. Lancet.

